# Synthesis and Translation of Viral mRNA in Reovirus-Infected Cells: Progress and Remaining Questions

**DOI:** 10.3390/v10120671

**Published:** 2018-11-27

**Authors:** Guy Lemay

**Affiliations:** Département de microbiologie, infectiologie et immunologie, Université de Montréal, Montréal, QC H3C 3J7, Canada; guy.lemay@umontreal.ca; Tel.: +(514)-343-2422

**Keywords:** reovirus, transcription, RNA capping, translation, protein synthesis, PKR, interferon

## Abstract

At the end of my doctoral studies, in 1988, I published a review article on the major steps of transcription and translation during the mammalian reovirus multiplication cycle, a topic that still fascinates me 30 years later. It is in the nature of scientific research to generate further questioning as new knowledge emerges. Our understanding of these fascinating viruses thus remains incomplete but it seemed appropriate at this moment to look back and reflect on our progress and most important questions that still puzzle us. It is also essential of being careful about concepts that seem so well established, but could still be better validated using new approaches. I hope that the few reflections presented here will stimulate discussions and maybe attract new investigators into the field of reovirus research. Many other aspects of the viral multiplication cycle would merit our attention. However, I will essentially limit my discussion to these central aspects of the viral cycle that are transcription of viral genes and their phenotypic expression through the host cell translational machinery. The objective here is not to review every aspect but to put more emphasis on important progress and challenges in the field.

## 1. Introduction

Mammalian orthoreoviruses, hereafter referred to as reovirus, are members of the Reoviridae family, a large family of viruses that have three major characteristics in common. Firstly, their genome is made of a certain number of double-stranded RNA segments. Secondly, they all possess a naked capsid made of concentric protein layers. Finally, the genome of these viruses is never completely released but rather transcribed by viral enzymes retained inside a viral particle in the cytoplasm of the infected cell. Mammalian orthoreoviruses have been instrumental in understanding various aspects of Reoviridae replication, although the last decade has seen considerable progress in the understanding of other family members. Some of this work, especially on rotaviruses and orbiviruses, will occasionally be referred to when equivalent data are not available for reovirus.

Despite recent accumulating evidence of transmission to humans or domestic animals of new pathogenic strains [1,2,3], reovirus is generally believed to be almost not pathogenic in humans. Nevertheless, they have been extensively used as a model system by prominent figures of late 20th century virology as Bernard Fields, William Joklik and Aaron Shatkin to name just a few. In the last two decades, these viruses have again attracted the attention of the scientific community since they are among the few naturally oncolytic viruses currently under clinical trials [4,5] and they have been recognized as orphan drugs by the American Food and Drug Administration and the European Medicines Agency to treat some forms of cancers (e.g., gastric, pancreatic, peritoneal, ovarian). More recently, they have been used once again as a model system to support the idea that viruses could contribute to breaking of tolerance to food antigens such as gluten. This could lead to celiac disease and largely asymptomatic reovirus infection appears to be clinically correlated with the disease [6].

## 2. The Reovirus Particle

The viral genome consists of ten double-stranded RNA segments or genes named as small (S1 to S4), medium (M1 to M3) or large (L1 to L3) [7]. Each viral gene in mammalian orthoreoviruses harbors identical GCUA sequence at the 5′-end and UCAUC at the 3′-end; other related viruses such as pteropine reovirus also harbor conserved sequences that may however differ. The viral genome and resulting mRNA is not polyadenylated but the plus strand of the viral genome, and at least part of the viral mRNA, harbors a cap structure; this will be further discussed below. The viral capsid is made of 8 proteins while 4 nonstructural proteins are made during the viral replication cycle. The capsid proteins are assembled as an outer capsid and an inner capsid or core; turrets present at the 12 vertices of the capsid structure are made of a single viral protein assembled as homopentamers and serve as anchors for the viral cell-attachment homotrimeric protein [8].

## 3. Brief Overview of Reovirus Replication Cycle 

**Figure 1:**
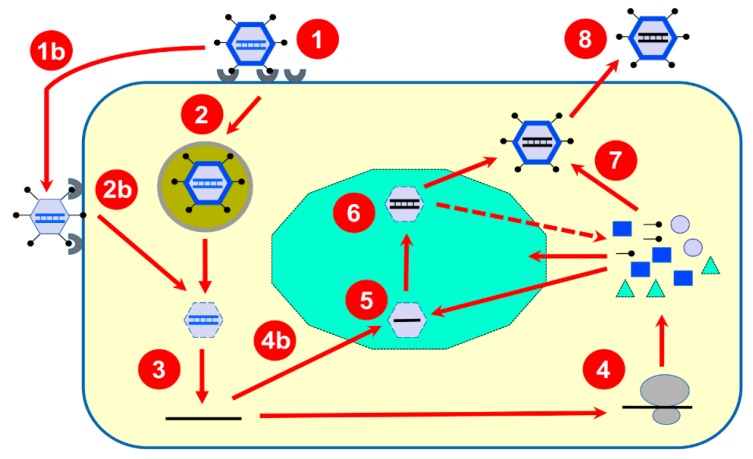
Summary of the reovirus multiplication cycle. Upon binding to different host-cell receptors (**1**), the reovirus virion containing the double-stranded RNA genome (in blue) is internalized by endocytosis (**2**) and transported towards late endosomes (**2**); they are then partially disassembled to produce transcriptionally active cores that are released in the host-cell cytoplasm (**3**). Alternatively, due to the action of extracellular proteases, partially uncoated virions known as infectious subviral particles (ISVPs) are generated (**1b**) and can bypass endocytosis and penetrate the host-cell membrane (**2b**) also leading to transcriptionally active cores in the cytoplasm (**3**). These “parental” cores possess all required enzymatic activities to synthesize and release multiple copies of capped messenger RNA (in black) synthesized from the ten viral double-stranded RNA genome segments as a template. Released mRNAs are translated by the cellular machinery (**4**) to generate both structural and nonstructural proteins; alternatively, some copies (**4b**) will be recruited to viral inclusions (in green) made by viral proteins. One copy of each 10 viral mRNAs will be packaged by viral inner capsid proteins to form intermediate structures (**5**) where synthesis of the second strand will take place to generate newly formed double-stranded RNA genomes retained in “progeny” particles (**6**). These, more abundant particles, in turn, are believed to be responsible for the synthesis of the bulk part of viral mRNA responsible for viral proteins synthesis (as indicated by the dotted arrow). Outer capsid proteins will be added (**7**) to finalize viral particles assembly before release of fully assembled virions (**8**).

An overall review of the early events, leading to the release of the core structure in the cytoplasm, was published in the last few years [9] and additional information could be found in the recent book chapter of *Field’s Virology* [7]; a schematic overview of the viral replication cycle is also presented on Figure 1.

Briefly, following host-cell binding to different cellular receptors (e.g., sialic acids, GM2 ganglioside, JAM-I, Ngo-R, B-integrins), the reovirus virions are internalized by clathrin, or possibly caveolin-based, endocytosis. Following endocytosis, stepwise proteolytic digestion of the outer capsid leads to acquisition of a membrane-penetrating ability to cross the late endosomal membrane. Alternatively, partially uncoated particles, known as infectious subviral particles (ISVPs), can bypass endocytosis and gain access to the cell’s interior directly through the plasma membrane. The remaining outer capsid proteins will be eliminated either during or after translocation to the host-cell cytoplasm, to generate the transcriptionally active core structure. The uncoating efficiency by the combined effect of lysosomal enzymes and autocleavage by the outer viral capsid μ1 will thus contribute to the efficiency of transcription initiation. In addition, a cellular chaperone is apparently involved in the later steps of uncoating [10]. Cleavage products of the remaining outer capsid proteins are finally released, and the viral cell attachment protein (σ1) is removed from the λ2 pentameric channel, opening it for viral mRNA extrusion while transcription occurs. The presence of the σ3 and μ1 heterohexamer forming the outer capsid is sufficient to inhibit the transcription of full-length mRNA molecules [11]. However, a recent report also suggests that the amount of σ1 protein, or efficiency of its release from the virion, could be a limiting factor to viral mRNA export from the transcriptionally active particle [12]. Interestingly, various reports indicate that different viral isolates can harbor different amounts of σ1 spikes [12,13,14,15,16], while an earlier report even suggested that as few as three copies of the trimers are sufficient for normal infectivity on fibroblasts in tissue culture [17]. There is thus probably a balance between the amount of σ1 required for optimal infectivity and that required for its most efficient removal allowing synthesis and release of viral mRNA. 

## 4. Progress in Our Experimental Approaches

Over the past 25 years, the use of site-directed mutagenesis approaches, combined with the development of more efficient nucleic acid sequencing techniques, has contributed to the understanding of viral proteins functions. Essentially, these studies relied on different approaches of in vitro characterization using proteins recovered from various expression systems. Cryo-electron microscopy combined with the structural analysis of proteins by X-ray crystallography has also increased our knowledge of viral particles structure [18]). But it is only as recently as 2007, that a T7 plasmid-based reverse genetics technique finally allowed to alter with relative ease the genome of reovirus and thus study the effect of predetermined changes on the viral multiplication cycle [19,20,21]. More recently, high-throughput approaches have also been used to extensively look at the effect of reovirus infection on either the cellular proteome [22,23,24,25] or transcriptome using microarrays [26,27,28,29,30,31] or more extensive RNA-seq approaches [32,33]. Altogether, these approaches are likely to shed new light on the complex interactions between the host cell and the virus, as discussed below.

## 5. Synthesis of Viral mRNA

### 5.1. Transcription of Viral dsRNA Genome to mRNA by Inner Core Enzymes

The inner capsid with its turrets of λ2 pentamers forms the core, active at the transcriptional level and capable of producing viral messenger RNA with a methylated cap-1 structure. Viral mRNA is then released from the cores through the turrets, giving rise to impressive images in electron microscopy [34]. The λ3 protein had already been identified as the viral polymerase in the 1980s, using gene reassortment to identify the gene responsible for the pH optimum of transcription [35]. Biochemical and ultrastructural studies have confirmed beyond doubt that this protein is responsible for the transcription of the viral dsRNA genome [36,37,38]. Detailed discussion of the structure of the RNA polymerase can be found in an excellent book chapter [39] and is clearly outside the scope of the present review. However, it should be mentioned that the protein harbors a consensus GDD motif and general thumb, palm, and priming loop structures common to many viral RNA polymerases, as also recently reviewed [40,41]. The reovirus polymerase does not need a primer per se to initiate de novo RNA synthesis but conserved sequences present at the end of the viral genome are likely required for recognition prior to initiation of polymerization; the second conserved nucleotide on the template strand is especially important to recruit the polymerase allowing initiation of mRNA synthesis from the priming nucleotide. Approximately 12 copies of λ3 per virion are present under the inner capsid close to the base of each turret. Ultrastructural evidence indicates that the newly synthesized mRNAs cross the outer capsid protein layer through a channel at the interface of two λ1 subunits, then through the channel in the middle of the λ2 pentamer [38]. The minus strand of the double-stranded RNA enters the λ3 polymerase through a first channel in the enzyme structure and exit through a second channel; the mRNA synthesized and the nucleotides enter through two other channels in the enzyme structure. The 5′-cap structure on the plus strand of the double-stranded RNA is tethered to λ3 and this is believed to be important for the initiation of the transcription. As for most viruses, the nucleic acid concentration inside the viral particle is high, most recent estimates suggest the concentration to be as high as 400 mg/mL [42]. In Reoviridae, however, the viral genome remains in the viral particle’s interior where high viscosity conditions are present thus likely limiting movement of transcriptional complex but still allowing RNA movement [43]. The currently accepted model is sometimes referred to as a “fixed-polymerase-moving-template” situation. In different dsRNA viruses, there is evidence that the RNA is well ordered inside the capsid and it is likely that each genomic segment is associated with a single transcription complex [44,45,46,47,48] and currently available data indicate that it is also likely to be the case in reovirus [36]. Interestingly, in the non-turreted rotavirus, transcription of each gene was recently shown to be specifically associated with one transcription complex and release of each corresponding transcript was shown to occur through one given channel of the transcribing particle [49]; hopefully, similar experiments will be performed for other members of the Reoviridae, such as mammalian reovirus, in the near future.

As was previously discussed [50], transcription of the reovirus genome to single-stranded mRNA is well known to be conservative. The positive and negative strands forming the genomic double-stranded RNA are kept intact in the capsid and only the newly synthesized plus-strand mRNA progeny is extruded from the core to the cytoplasm. Ample evidence suggests that relatively long sequences are required for adequate function of the viral genomic segments and/or mRNA [51,52,53,54,55]; some of these, however, are rather required for packaging or synthesis of the negative strand of the RNA genome, these aspects will not be further discussed herein.

The reovirus genome is among the biggest RNA genome at approximately 24 kbp; only coronaviruses have a lengthier (although single-stranded) RNA genome. RNA polymerases do not possess proofreading ability hence the high mutation rate of RNA viruses that may limit their size to allow maintenance of viable genomes through time. Interestingly, coronaviruses were recently shown to possess such an activity; although the required exonuclease activity is encoded by a protein distinct from the polymerase itself [56]. There is no evidence that reovirus possesses such a proofreading ability although this could certainly deserve further study. The replication strategy where the genome is repeatedly used to generate the first strand of new genomes, avoiding a classical semi-conservative strategy, is also a factor that should significantly reduce accumulation of replication errors. To our knowledge there has been no precise measurement of mutation rates in reoviruses, although in the dsRNA phage phi6 (ϕ6), frequency of nucleotide substitution appears lower than in other RNA viruses [57]. However the ϕ6 replication strategy is slightly different by release of the plus strand of the viral genome rather than its de novo synthesis [58]; its genome is also much smaller, making comparisons difficult.

The necessity of a viral helicase to unwind the dsRNA genome during the transcription of the reovirus genome remains controversial. It has been proposed that the cap-binding site on the λ3 polymerase itself is sufficient to ensure strand dissociation [37] but it does not seem that direct experimental evidence is available to support this view. Interestingly, crystal structure of rotavirus polymerase does also show a cap-binding site [59] while the bluetongue virus polymerase does show a cap dependency [60], at least for copying of a single-stranded RNA template in vitro. This suggests that these polymerases all use a cap structure to ensure template binding and/or unwinding. Although the reovirus polymerase was shown to possess a polymerizing activity by itself in vitro, it appears to be limited and unable to actually transcribe dsRNA [61]. It is unclear if this is solely due to inappropriate experimental conditions or to the actual necessity of other viral proteins for a full ability to transcribe the viral genome. Two viral core proteins, μ2 and λ1, harbor consensus motifs consistent with a helicase activity, as well as expected affinity for nucleic acids.

Several arguments support the idea that the μ2 protein present in approximately 24 copies at the base of the turrets, acts as a co-factor for the polymerase and thus possibly as a helicase. Temperature optimum of transcription, sensitivity to a guanine nucleotide synthesis inhibitor, and optimal temperature of core-associated ATPase activity were all assigned to the μ2-encoding M1 gene [62,63,64]. However, indirect effects cannot be excluded, especially since a role of μ2 optimal assembly of viral genome and particles was observed [65,66,67].

The λ1 protein also exhibits similar motifs and biochemical properties, acting as an ATPase/helicase in vitro [68,69]. As μ2, it is part of the core but, unlike μ2, it is also a major structural component of it. The position of the catalytic sequences in the N-terminal region intracapsidic portion of the protein, a region that is dispensable for core assembly [70] is, however, consistent with a possible role as a helicase. The structure of this part of the protein appears to be disorganized and possibly cleaved during the capsid reorganization that leads to transcriptional activation of the core particle [36,43,71]; this was interpreted as either precluding or suggesting its role in transcription. For now, no published study has clearly established or ruled out the importance of the catalytic region of λ1 for the synthesis of viral RNA in the context of a viral infection. Recent data showing that λ1 can contribute to the control of interferon induction [72] suggest additional role(s) for the well-known affinity of the protein for nucleic acid [73,74] and/or for its enzymatic activity.

Results obtained in the 1980s and previously reviewed [50] indicated that the transcriptional frequency of the different genes is indirectly proportional to their length when performing in vitro transcription reaction using purified cores. This is consistent with the idea that each dsRNA segment in the viral capsid is transcribed by a given molecule of RNA polymerase acting at the same speed on the template, as previously mentioned for the recent work on rotavirus [49]. Other earlier reports indicating that the ratio of the ten reovirus mRNAs in the infected cells differs from those obtained in vitro are difficult to reconcile with such a model and the mechanism involved, if any, remains unclear. It will be of interest to clarify this point using modern approaches for precise RNA quantitation, such as quantitative RT-PCR or RNA-seq analysis. Another aspect that remains unexplained is the previously discussed observations [50] showing that only four viral genes are transcribed at very early times post-infection, and that protein synthesis is required to relieve this apparent blockage of transcription on the six remaining genes. In the absence of additional data, it is difficult to assess if this transcriptional regulation actually takes place considering the technical challenges of the hybridization methods used to measure the different viral transcripts at the time.

### 5.2. Synthesis of the Cap Structure on Viral mRNA

**Figure 2:**
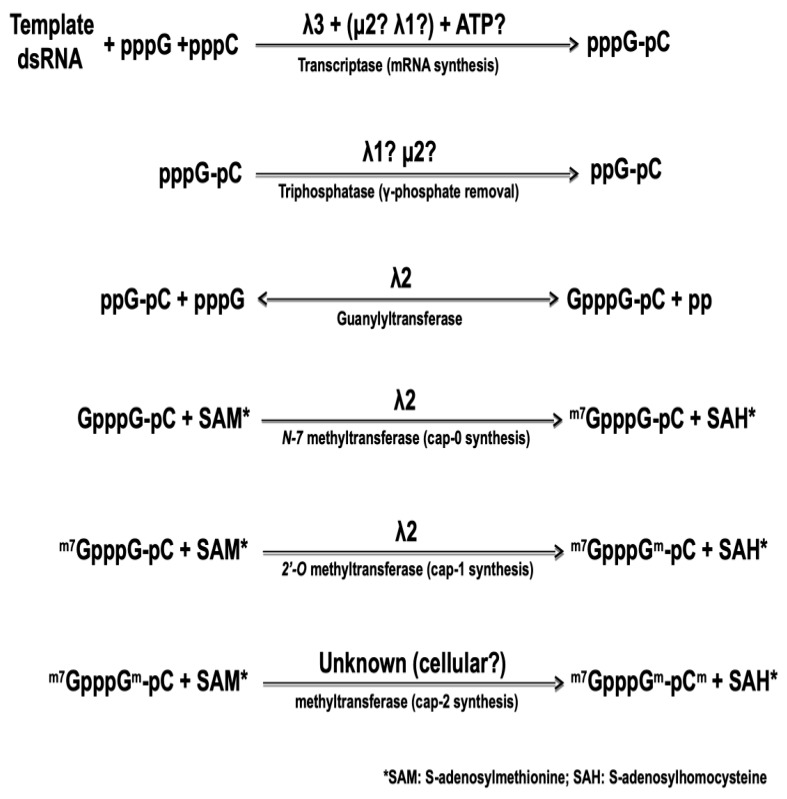
The different steps in the synthesis of capped reovirus mRNA by reovirus core. The nature of the different viral proteins involved is indicated, as described in the text.

The 5′-end of the viral mRNA harbors a cap structure that is synthesized by sequential enzymatic steps that are essentially identical to those of cellular mRNA cap synthesis, although catalyzed by viral core-associated enzymes, as summarized on Figure 2, below. The nature of the viral protein responsible for the first step of cap synthesis, namely the RNA triphosphatase (or phosphohydrolase) removing the first phosphate at the 5′-triphosphate end of the newly synthesized RNA, also remains controversial. Again, both μ2 and λ1 exhibit such an activity in vitro [75,76] with very similar enzymatic parameters. In both cases, the affinity for the substrate as measured by the Michaelis–Menten kinetics was shown to be 10-fold better for 5′-triphosphorylated RNA than for free nucleotides. It thus remains possible that one or the other of the two proteins first acts as an NTPase/helicase but is then preferentially active on the nascent RNA substrate.

Amino acids residues essential for the μ2 catalytic activity are necessary for virus recovery in a complementation assay where expression of the wild-type protein encoded by the infecting virus is inhibited by siRNA [77,78]; however, the exact step of the viral replication cycle that is affected remains unclear. Furthermore, to our knowledge, similar experiments have not been performed yet with λ1. At this point, there is thus no direct evidence to clearly assign the RNA triphosphatase activity to either of these proteins. Recent structural data on cypoviruses and aquareoviruses, other turreted dsRNA viruses of the Reoviridae family, indicated that the analog of μ2 is positioned inside the core at the basis of the turret and in interaction with the viral polymerase. However, its position precludes an interaction with the 5′-end of the nascent viral mRNA and this appears to rule out the possibility that it acts as the RNA triphosphatase [48,79]. To our knowledge, similar data are not yet available for reovirus.

In contrast, the guanylyltransferase forming the *5′-5* triphosphate link between the first nucleotide and a GDP molecule from GTP, in the second step of cap synthesis, has been known for more than 25 years. The core-associated λ2 turret protein possesses this activity by itself [80,81,82]. Following the formation of this link, the so-called cap 0 structure is formed by the addition of a methyl at position *N-7* of the transferred GMP. Addition of a *2′-O* on the first nucleotide of the mRNA itself then forms the cap 1 structure. Both methyltransferase activities are also clearly associated with λ2 and correspond to the regions around amino acids 420–720 and 800–1030 approximately; however, there is still a controversy concerning the role of each region in forming the cap 0 and cap 1 structure by adding the corresponding methyl group. The position of the two methyltransferase domains has led to suggest that separate molecules of the λ2 turrets are involved in the consecutive addition of the two methyl groups [36]. This idea is consistent with the guanylyltransferase activity observed with purified λ2 while methyltransferase is not [81], suggesting that formation of a higher-order complex is necessary for this latter activity. It was also postulated that the order of the three domains on the protein reflects the order of the reactions, the last methyltransferase domain being responsible for the *2′-O* methylation forming cap 1 structure [36]. However, detailed sequence homology and structure analysis have rather led other investigators to propose that the order is in fact the opposite, the most amino-terminal domain being rather responsible for the *2′-O* methylation [83]. Interestingly, recent crystallographic data of a cypovirus, another member of the Reoviridae harboring a turret similar to that of reovirus, revealed a similar organization where the *2′-O* methyltransferase is believed to be located after the *N7*-methyltransferase on the linear structure of the protein [84]. These data are in agreement with a model where the RNA travels to be in contact with a least two, and possibly three, molecules of the capping enzyme [38]. Our recent work [85], showing that an amino acid substitution in the first methyltransferase domain leads to increased interferon sensitivity of the virus supports this idea. Increased induction and/or sensitivity to interferon have previously been assigned to an impaired *2′-O* methylation for different viruses [86,87,88]. Engineering of mutant viruses harboring amino acids substitutions in either methyltransferase domain could help to clarify which domain is actually responsible for each step of methyl addition to the cap structure.

In infected cells, in contrast to the situation observed for mRNA transcribed from purified cores in vitro, it has been reported that a certain proportion of the mRNA molecules harbors a cap 2 structure, with the second nucleotide of the mRNA also methylated. This has been reviewed in the past but evidence remains limited [89,90]. If a proportion of reovirus mRNA is actually harboring a cap 2 structure, a cellular activity must be involved in the addition of this third methyl group. The recent discoveries of cytoplasmic capping activities, and especially the cytoplasmic presence of the cellular methyltransferase involved in cap 2 formation [91,92,93,94], does support this possibility. Interestingly, it has been observed that the presence of interferon could preclude the synthesis of this cap 2 structure although, this does not seem to have been further established since these early reports [95,96]. An effect of interferon on relative amount of methyl donor to methyl acceptor molecules (*S*-adenosylhomocysteine to *S*-adenosylmethionine) has also been reported [97]. Clearly, it will be of interest to re-examine the effect of interferon on cellular and viral mRNA capping and on the presence or intracellular distribution of the cellular enzymes involved.

The work carried out in the laboratory of Dr. Stewart Millward at McGill University in the 1980s suggested that a transition in the nature of the viral mRNA 5′-terminal end occurs during infection. Messenger RNA transcribed by parental virus particles are capped while in progeny particles the enzymatic activities responsible for cap synthesis appear to be either absent, inactive or masked [98,99,100]. There have been reports of a transient lack of the λ2 turrets on the progeny particles, as previously discussed [50,89,90]. Although later observations failed to support this presence of particles devoid of λ2 in a wild-type virus, they nevertheless indicated that viral cores can be assembled without the turret in a thermosensitive mutant [101]. Also, the initial suggestion that capping enzymes were masked by the presence of nonstructural inclusion protein μNS on the particles [89] was not supported by further studies showing that this does not actually interfere with the capping activities [102]. The mechanism behind the apparent “masking” of capping activity thus remains elusive; the development of new powerful ultrastructural imaging techniques could allow to clarify the exact structure of the viral particles at different times during the multiplication cycle, in order to answer this question. It should also be mentioned that the RNA synthesized from viral particles in vitro, harbors a diphosphate end, when produced under conditions favoring RNA production without the cap structure. This is achieved in absence of the methyl donor (SAM) and in the presence of the methyl acceptor (SAH) to prevent the methylation of the cap structure. The reversal of the guanylylation reaction of these unmethylated cap structure is also favored by adding an excess of the reaction product (sodium pyrophosphate) to the reaction. In contrast, the late viral RNA extracted from infected cells exhibits a monophosphate 5′-end, suggesting action of a phosphatase, viral or cellular, a point that has never been further examined. We still do not know the nature of the factor(s) involved, if any. Avoiding the presence of a diphosphorylated 5′-end is a strategy used by other viruses to prevent activation of antiviral program of the innate immune response in infected cells, through recognition by RIG-I or by IFIT1 (Retinoic acid-Inducible Gene I or Interferon Induced Protein with Tetratricopeptide Repeats 1) [103,104,105,106,107]. The importance of the innate immune response in the control of protein synthesis will be further discussed in Section 7.4. Strikingly, a study published in 2013 [108] suggests the presence of a proportion of uncapped rotavirus mRNA late in infection that is recognized by the innate immune system. This again suggests that capping of rotavirus or reovirus mRNAs is probably incomplete, at best, and this could play an important role in the ability of the innate immune system to recognize and control the infection.

## 6. Impact of Reovirus Infection on Cellular mRNA

Earlier work has suggested that reovirus infection could inhibit the transcription of cellular mRNAs [109] but, to our knowledge, this aspect was not further investigated. Its significance remains difficult to assess since decreased transcription was observed concomitantly with decreased protein synthesis and only for serotype 2. The fact that reovirus is a cytoplasmic RNA virus harboring all its transcriptional/capping machinery could lead to the impression that it should not affect nuclear processes. However, there is accumulating evidence of cytoplasmic RNA viruses that either take advantage of, or affect nuclear processes [110,111,112,113]. For example, in rotavirus, sequestration of the poly(A) binding protein to the nucleus is believed to contribute to decreased levels of cellular mRNA available for translation in the cytoplasm [114,115] but, to our knowledge, this has never been examined for reovirus.

At least three reovirus proteins (σ3, σ1s and μ2) have been previously observed, at least in part, in the nucleus of either transfected or infected cells [78,116,117,118,119,120]. Although the σ proteins are probably small enough to be partly located to the nucleus by diffusion, other reovirus proteins will require a nuclear localization signal. Interestingly, μ2 and λ3 are predicted to harbor such a signal. Our recent work, using mass spectrometry, allowed to confirm a strong nuclear enrichment of μ2, and a significant enrichment of the viral RNA polymerase λ3 [32]. The role of nuclear λ3, if its nuclear presence is confirmed, remains undetermined but, interestingly, it was recently observed by us and others that reovirus infection significantly affect the pattern of alternative splicing of a certain number of cellular pre-mRNA [32,33]. Although μ2 appears to be at least partly involved [33], a possible involvement of λ3 will be somewhat reminiscent of the poliovirus polymerase that was also found in the nucleus and shown to alter the splicing machinery of the infected cells [121]. A better understanding of alternative splicing in reovirus biology appears as a whole new research avenue to be explored.

Although the presence of other nuclear viral proteins could not be excluded in this mass spectrometry assay, the absence of σ3 could possibly result from artefactual nuclear localization in previous studies that used overexpression in transfected cells. This should be further investigated at different times post-infection to rule out transient nuclear presence at earlier times post-infection. It should be mentioned that previous results have shown that the presence of μ1, not surprisingly, interferes with σ3 nuclear presence [116,117] and the balance between free σ3 and μ1 at different times post-infection could thus alter the ratio between cytoplasmic and nuclear σ3, if any, and may have its importance on synthesis or maturation of cellular mRNA.

In addition to mRNA synthesis and splicing, recent data also indicate that certain virus strains affect the stability of certain cellular mRNA [122], the exact significance of this observation and the viral protein involved remains to be established but appears to be somehow linked to the capacity of the viral strain to inhibit synthesis of host-cell proteins.

## 7. Protein Synthesis in Reovirus-Infected Cells

### 7.1. Structure of Reovirus mRNA

**Figure 3:**
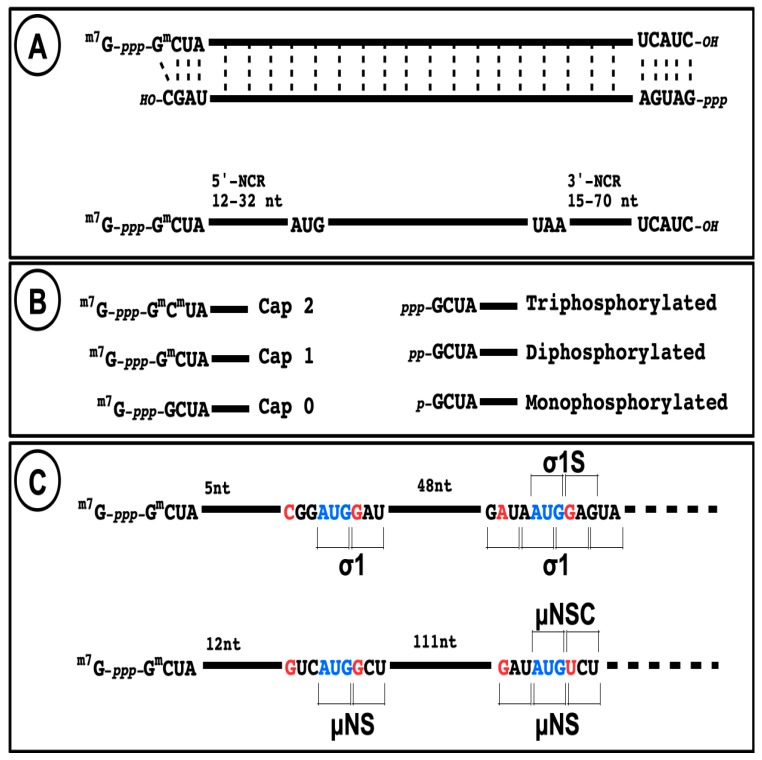
Structure of reovirus RNA. (**A**) The structure of a viral dsRNA genome segment is presented with the different 5′ and 3′-terminal structure and conserved sequences at both ends, as observed for mammalian orthoreoviruses. The structure of a typical mRNA is presented with the length of both 5′ and 3′ non-coding regions. (**B**) Schematic representation of the different possible capped and uncapped 5′-end on mRNA. (**C**) Schematic representation and nucleotide sequence at the two translational initiation sites on s1 and m3 mRNAs.

The 5′-untranslated region (or non-coding region, NCR) of all ten species of reovirus mRNA is relatively short, as indicated in Figure 3, below. Some of them are even under the 20-nucleotide length considered as minimal for efficient translational initiation at the 5-proximal initiation codon (AUG) following ribosomal scanning [123]; however, the so-called TISU consensus sequence (Translation Initiator of Short 5′-UTR) [124] is not present on reovirus mRNA.

In the absence of other evidence, it should be assumed that initiation of translation on reovirus mRNA follows the classical ribosomal scanning mechanism, initiating at the first AUG codon. Although it has been suggested that the μ2 protein is rather synthesized from the second AUG codon on the messenger RNA [125], this was thereafter clearly ruled out [126,127]. In mammalian orthoreoviruses, each viral gene also encodes only one primary translation product except for S1 and M3. The s1 mRNA encodes both σ1 and a second small nonstructural protein (σ1s) in a second reading frame bypassing the first initiation codon (Figure 3), as well established by numerous studies [128,129,130]. Interestingly, this was among the first mRNA known to be translated on an alternative open reading frame in eukaryotic cells. This strategy is now known to be relatively frequent on viral mRNA, and recently shown to be potentially more frequent than expected on cellular mRNAs [131]. The m3 mRNA encodes both μNS and a second shorter form (μNSC) by initiating translation at a second in-frame initiation codon [132,133] (Figure 3). To date, there is no evidence that alternative open reading frames are used on other mammalian reovirus mRNAs; only one downstream open reading frames beginning with AUG can potentially encode a peptide longer than 100 amino acids and the surrounding context does not correspond to a favored Kozak’s consensus sequence, making it unlikely that it is actually used. However, it is not possible to completely exclude that other mechanisms could allow alternative initiation. In fact, other orthoreoviruses, such as avian or reptilian reoviruses, were shown to encode up to three different polypeptides on the same mRNA while the alternative translational initiation mechanisms used remain incompletely understood [134,135].

In contrast to the differences in transcriptional efficiency, it appears that the idea that the different viral mRNAs vary in their translational efficiency is well established [50]. Although other factors may well contribute, an extensive folding of the 5′-untranslated region could decrease the efficiency of translation (such as in the s1 mRNA) while shorter, less structured, 5′-end results in increased translation (such as in s4 mRNA), at least in vitro [136]

Most eukaryotic mRNAs also possess a 3′-end poly(A) tract that is involved in both stability of the mRNA and its translation through recognition by the poly(A)-binding protein in the so-called closed-loop model of translational initiation [137,138,139]. In contrast, as previously mentioned, reovirus mRNAs, as all mRNA of viruses from the Reoviridae family, are not polyadenylated at their 3′-end. In vitro translation studies have established that reovirus mRNA translation is not sensitive to inhibition by free poly(A) nor stimulated by addition a poly(A) tract at their 3′-end [140]. This indicates that they are not dependent on poly(A) binding protein. In rotavirus, despite some controversies, most recent evidence support the idea that a nonstructural viral protein somehow substitute for cellular poly(A)-binding proteins [141,142]. There is no evidence to date of a similar function among reovirus proteins.

### 7.2. Synthesis of Viral Proteins during the Viral Multiplication Cycle

As mentioned earlier, viral mRNAs quantitatively responsible for the synthesis of the bulk part of the viral proteins are likely devoid of both the 3′-poly (A) tail and the 5′-cap structure, important structures for both stability and translation of eukaryotic mRNAs. More than 30 years ago, the reovirus protein σ3 was shown to stimulate translation of viral mRNAs, but only those devoid of a cap structure. This protein behaves more or less like a translation initiation factor and was found associated with ribosomes [140,143]. Additional work indicated that the protein is released by high-salt treatment known to release initiation factors, but not by treatment that will separate the two ribosomal subunits (EDTA) or remove the mRNA template from the ribosomes (micrococcal nuclease) [144]. Furthermore, using in vitro translation system, there were indications that the protein is released from the ribosomes under conditions in which initiation of translation is blocked, while it accumulates on the ribosome when initiation is allowed but elongation is prevented [144]. Similar reports of viral proteins apparently acting as initiation factors of protein synthesis have been reported in the last ten years for RNA and DNA viruses belonging to diverse virus families [137,145,146], including bluetongue virus as another Reoviridae [147]. Interestingly, a recent study revealed that the translation of uncapped infectious bursal disease virus RNA, another dsRNA virus of the Birnaviridae family, relies on the presence of a viral protein that may act as a substitute for the translation initiation factor eIF4E [148]. This suggests that the use of a viral protein to stimulate viral uncapped mRNA translation could be a mechanism shared by different dsRNA viruses.

The association of σ3 with ribosomes may also explain the reported presence of σ3 in the nucleus [116,117], since preliminary observations suggest a possible nucleolar localization when wild-type σ3 was expressed by itself (unpublished observations). The ability of the protein to bind dsRNA does appear as a prerequisite for this nuclear presence [116]. However, despite the presence of a putative nucleolar localization signal, this should be viewed with caution in the absence of more direct evidence in infected cells. Overall, this should be further examined in the context of viral infection, at different times post-infection, as the importance of the nucleolus in the multiplication of various viruses has now been established [149,150,151,152,153]. The relative importance of σ3 in stimulating translation of uncapped viral mRNA and/or inhibiting the translation of cellular mRNAs remains unclear, as will be discussed in the next section.

It was also recently reported that another viral protein, the σ1s protein, contributes to efficient synthesis of viral proteins, at least in some cell types [154]. The mechanism involved is unknown although σ1s apparently also contributes to the control of the antiviral activity of interferon [155]. The importance of interferon in the control of protein synthesis is further discussed in Section 7.4.

Another recent groundbreaking observation also suggests that, contrary to what was generally assumed, the translational machinery is present and active in viral inclusions [156], as previously observed in large cytoplasmic DNA viruses that also possess a complete transcriptional machinery [157,158]. This could allow to secure translation of viral proteins at the site of viral mRNA synthesis and viral assembly, and could contribute to shielding viral nucleic acids from recognition by the innate immune system. Surprisingly, other studies do not indicate the presence of ribosomes in the inclusions [159]. Overall, it appears that clarification of the involvement of the viral inclusion during reovirus mRNA translation will require further investigation.

### 7.3. Impact of Reovirus Infection on Translation of Cellular mRNAs

Upon infection of the classically used L929 mouse fibroblasts in culture, a significant decrease is observed in synthesis of cellular protein, this inhibition being variable among viral strains [109,160]. There has been a controversy in the literature concerning the possibility that reovirus mRNA and cellular mRNA are in competition for a limiting factor in the infected cells. This was postulated to be cause for inhibition of host-cell mRNA translation, due to excess viral mRNA [161,162]. This hypothesis has been previously discussed in greater details [50] and various arguments against this possibility have been presented over the years [163,164,165]. In contrast, a series of experiments suggested that late reovirus-infected cells present a decreased ability to support cap-dependent translation of either cellular or viral mRNA [100,163,166,167]. The exact alterations of cap-binding proteins in reovirus-infected cells remain to be more firmly established. It was observed that the addition of a monoclonal antibody directed against the 24 kDa eIF4E cap-binding protein has no effect on translation in cell-free extract prepared from infected cells, in contrast to the inhibition of cap-dependent translation in uninfected cells [168]. This supports the idea of an altered function of cap-binding proteins upon reovirus infection. A decreased amount of crosslinking to radiolabeled cap structure was also observed for a certain number of proteins in infected cells [167]; however, it does not seem that the phosphorylation of eIF4E is altered, in contrast to the situation observed in inhibition of cap-dependent translation in cells infected with members of the Picornaviridae [169]. Clearly, this should be further investigated now that specific reagents and approaches such as specific antibodies for immunoblotting, RNA interference, gene knockout and mass spectrometry are available to examine more closely each component of the translational machinery. Also, there is clearly a functional difference between uncapped-diphosphorylated reovirus RNA (ppG-terminated) synthesized in vitro and in vivo-synthesized monophosphorylated RNA (pG-terminated). While translation of ppG-terminated RNA is resistant to addition of a cap analog translational inhibitor (^m7^GTP), as expected, pG-terminated RNA was surprisingly found to remain sensitive even in infected cell lysates [163]. This further stresses that earlier competition experiments using in vitro-synthesized RNA should be viewed with caution. It will be interesting to revisit this issue by taking advantage of various chemical inhibitors now available.

One might thus think that a transition from cap-dependent to cap-independent (and virus specific?) does actually occur during the viral multiplication cycle of various dsRNA viruses. This transition could contribute to the release of viral capped mRNA from the ribosomes so that it can be used exclusively for the synthesis of double-stranded genome since it is well known that the first strand (by definition positive strand) of encapsidated viral double-stranded genomic RNA harbors a cap structure, as discussed in Section 5.1.

Gene reassortment analysis has suggested that the σ3 viral protein is involved in inhibition of host-cell protein synthesis; variation in the extent of this inhibition among viral strains apparently correlates with different subcellular distribution of σ3 [109,160,170]. The hypothesis is that the long-known ability of the protein to bind double-stranded RNA (dsRNA) could inhibit the cellular dsRNA-dependent antiviral protein kinase, PKR. The protein kinase PKR is a cellular, interferon-inducible, antiviral protein that requires dsRNA for its dimerization; the protein can then autophosphorylate becoming activated to phosphorylate different substrates, including translational initiation factor eif2α resulting in inhibition of protein synthesis [171,172,173]. The importance of PKR in the control of viral infection is supported by the fact that numerous viruses have developed one, or even many, factors to counteract its activity [146,171,174,175]. Not surprisingly, PKR is inhibited in vitro or in transfected cells in the presence of σ3, due to competition for dsRNA binding [170,176,177,178]. Reovirus σ3 can also substitute for known viral inhibitors of PKR to allow replication of such viruses as adenovirus, vaccinia virus and paramyxovirus [179,180,181,182]. Despite these different observations it remains controversial if PKR is involved, or solely responsible, for inhibition of host-cell protein synthesis during reovirus infection [27,165,183,184,185,186,187].

It has been proposed that upon infection with those viral strains exhibiting a less extensive colocalization of μ1 and σ3, synthesis of cellular proteins is spared [160,170]. This suggests that free σ3 is responsible, for the control of protein synthesis during infection. However, it should be emphasized that these different studies were performed in only one cell type and comparing different viruses that were not fully characterized. The use of isogenic strains harboring well-defined genetic alterations, as now possible through the use of reverse genetics, could better allow to understand the role of σ3.

Numerous arguments indicate that binding of σ3 to μ1 and dsRNA are mutually exclusive although binding sites may not directly overlap [188]. Interestingly, a σ3 thermosensitive mutant is unable to interact with μ1 at the restrictive temperature and, as a result, exhibits an increased ability to bind dsRNA and decreased susceptibility to interferon [117]. Much work has been devoted to the biochemical mapping of amino-acid motifs involved in dsRNA binding of σ3 and two different models, not necessarily mutually exclusive, have emerged [189,190]. In a first one, two basic motifs are found and are both involved although the second one, with a critical lysine, is most important [177,178,191,192]. In a later model, the RNA is proposed to be bound at the basic surface of a σ3 homodimer [193,194]. Recent data have actually confirmed the transition from σ3 homomultimer to structural σ3–μ1 heterohexamer by the action of cellular chaperonin [195].

When σ3 mutants are individually expressed in transfected cells, a correlation appears between their ability to bind dsRNA, their nuclear localization, and their ability to stimulate protein translation, most likely through PKR inhibition [117,170,177,178,190,191,192,193,194].

Altogether, a possible model thus emerges in which the σ3 protein is synthesized in excess compared to its μ1 partner and can thus exist in one of two forms: (i) as a dimer to exert a regulatory role in the control of translation regulation and (ii) as part of the heterohexamer complex with μ1 to form the bulk part of the outer capsid. The balance between these two functions will likely have an impact on viral multiplication and effect on its host cell.

### 7.4. Interferon, PKR, Stress, and the Regulation of Protein Synthesis during Reovirus Infection

A detailed discussion of the interferon response and its effect on viral replication goes beyond the scope of the present review. However, since it is becoming more and more evident that the activation of the interferon response consecutive to viral infection is of importance in the regulation of protein synthesis [196], it seems appropriate to briefly reflects on these aspects in the context of reovirus infection.

Recognition of viral signature following viral infection is followed by induction of interferon and interferon-stimulated genes. This could be followed by activation of the cellular protein kinase, PKR, consecutive to its homodimerization due to presence of viral double-stranded RNA, and inhibition of protein synthesis that could interfere with viral replication. In the case of reovirus, it does not seem that the genomic double-stranded RNA is exposed to the recognition by the innate immune system. The exact structure that is recognized as a pathogen-associated molecular pattern (PAMP) remains to be firmly established in the context of a viral infection. It has been clearly shown that diphosphorylated 5′-end present on the minus strand of the viral dsRNA genome is a potent PAMP recognized by RIG-I [197]; however, it is unclear if these will be actually exposed during the viral multiplication cycle. Obviously, if part of the mRNA molecules is devoid of a cap structure, this could also be recognized as non-self-RNA and induces interferon signaling. Extensive folding of some viral mRNA species could also be recognized. Finally, if some viral strains or viral mutants lack the ability to correctly methylate the viral cap structure, to make viral RNA as similar as possible to cellular mRNAs, this could lead to either induction or sensitivity to the antiviral response, as proposed for an interferon-sensitive reovirus mutant [85].

In NIH-3T3 cells, inhibition of PKR promotes viral replication, this observation has been at the origin of the idea of using reovirus as an oncolytic virus, since oncogenic Ras could also lead to inhibition of PKR [184]. Further work then indicated that oncogenic Ras can also inhibit interferon induction through negative regulation of RIG-I signaling involved in activation of the interferon response in the presence of viral RNA [198]. The presence of an activated Ras should thus promote replication, propagation and cell-killing activity of the virus in transformed cells. Accordingly, increased sensitivity to interferon of a mutant virus was shown to correlate with increased ability to discriminate between parental and Ras-transformed NIH-3T3 cells [199]. Since interferon response or signaling is known to be often deficient in cancer cells [200,201], a better understanding of reovirus determinants of induction of the interferon response or viral sensitivity to the induced response could clearly be of importance for a possible optimization of reovirus as an oncolytic virus [202,203].

**Figure 4:**
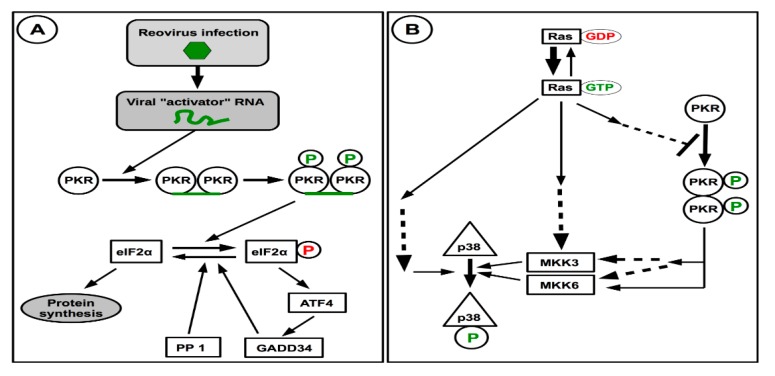
Activation of PKR and interaction with Ras activation and signaling towards p38. (**A**) In infected cells, recognition of a viral RNA results in PKR dimerization, autophosphorylation and activation. While activated PKR can directly phosphorylate eIF2α resulting in inhibition of protein synthesis, its indirect effect in the stress response by ATF activation could displace the equilibrium towards active unphosphorylated eIF2α to maintain protein synthesis. (**B**) Activation of the Ras oncogene that results in cell transformation can activate the p38 kinase by different routes. Activation of Ras also inhibits PKR activation by still unknown mechanism, this should result in limited PKR activation and consecutive p38 activation by this route, consecutive to viral infection. Dashed arrows indicate the presence of known or postulated intermediates.

In apparent contradiction, other data indicate that in the absence of PKR in murine embryonic fibroblasts, reovirus replication is actually reduced; this suggests that a certain level of cellular stress is required for efficient virus replication, at least in some cell types and with some viral strains [27]. This could be reconciled if one considers the multiple roles that PKR plays in the cell, normal or infected [171,173]. Although it is not possible to discuss this point in detail herein, an interesting observation is the fact that PKR can contribute to activation of the p38 pathway. Stimulation of the same pathway was previously shown to be involved in the stimulation of reovirus replication following Ras transformation [204,205]. Partial schematic representations of these pathways are presented on Figure 4.

Depending on prior status of the cell, a certain level of PKR activation could thus be beneficial to reovirus through activation of stress pathways leading to p38 activation. This could well vary between viral strains, depending on their sensitivity to the antiviral effect of PKR through eIF2α phosphorylation that may counteract this positive effect. These aspects in relation to the oncolytic activity of reovirus were most thoroughly discussed in a recent review in this journal [206].

Other evidence indicates the activation of PKR and eIF2α phosphorylation during viral infection, even though viral messenger RNA continue to be actively translated [185,186,207], as are some cellular, eIF2-independent, mRNA such as ATF4. Some of these data may seem contradictory and could be explained again by differences between viral strains, some of them probably having the ability to inhibit the antiviral activity of PKR at a level consistent with viral replication.

More recently, further progress has been made in understanding the regulation of protein synthesis in eukaryotic cells since the discovery of so-called “RNA granules” such as stress granules or processing bodies. These granules appear to be principally used by the cell to limit the use of the energy-expensive translational machinery by transiently sequestering RNA to be later translated or degraded. Accordingly, infection by a variety of viruses stimulates or inhibits the formation of these granules and in either case the result could promote or interfere with viral replication [208,209,210]. It has been shown that stress granules are actually formed during reovirus infection [27,211]; to our knowledge there has been no report of processing bodies formation, although this may just have been overlooked in reovirus studies. Later studies suggested that stress granules are formed at an early stage and probably dissociated to allow protein synthesis during the later stages of the infection [185,207]. Interestingly, some viral core and inclusion proteins were found in the stress granules [212] while some stress granules proteins could be trapped later in the inclusions [213]. Overall, this is consistent with a model in which viral mRNA is transiently stored in stress granules until inclusions are formed. This could contribute to avoid recognition by the host cell defense mechanisms, or allow compartmentalization of the synthesis of viral proteins late in infection, if it actually occurs in viral inclusions.

## 8. Final remarks

Despite the progress in our understanding of the reovirus multiplication cycle in the last 30 years, many questions remain on such basic aspects as how the viral genome is transcribed and how it is translated efficiently. Also, and even more strikingly, the effects of the virus on the cellular transcriptional, post-transcriptional, and translational machinery remain either controversial or poorly known. Some of the most striking questions are summarized in Table 1, below.

Such aspects could well become critical in our understanding of viral pathogenesis in potentially emerging reoviruses, as well as in our use of the virus as an oncolytic agent. The breakthrough of plasmid-based reverse genetics, as well as the development of new approaches based on transcriptomics, proteomics and gene-editing technologies, will likely accelerate our progress in the next decade. Hopefully, new investigators will likely pursue the work of the previous generations of virologists.

## Figures and Tables

**Table 1:** Remaining questions on synthesis and translation of reovirus mRNA

**Transcription of reovirus dsRNA genome**
•Is there a temporal regulation in the transcription of viral genes upon infection? •Is there involvement of helicase activity of μ2 or λ1 in transcription? •Are each segment of genomic RNA transcribed by a single transcription complex?
**Cap structure of reovirus mRNA**
•Is μ2 or λ1 acting as the RNA triphosphatase for viral mRNA cap synthesis? •Is cap2 structure present on viral mRNA and what is its role? •Is the capping activity absent, inactive or hidden in progeny cores?
**Impact of reovirus infection on cellular mRNA**
•Is transcription of cellular mRNA affected by reovirus infection? •What is the exact role of modified alternative splicing during reovirus infection?
**Synthesis of viral proteins**
•Is a viral protein acting as a surrogate of poly(A)-binding protein? •Is a translational operator present on viral mRNA? •Is protein synthesis modified to favor translation of uncapped viral mRNA? •What is the exact involvement of σ3 in the regulation of protein synthesis? •What is the mechanism allowing σ1s to stimulate synthesis of viral proteins?
**PKR, stress, and the regulation of protein synthesis during reovirus infection**
•Is PKR involved in inhibition of cellular protein synthesis? •What is the exact role of stress granules formation during reovirus infection?
